# Effectiveness of autogenic relaxation training in addition to usual physiotherapy on emotional state and functional independence of stroke survivors

**DOI:** 10.1097/MD.0000000000026924

**Published:** 2021-08-20

**Authors:** Deepak Thazhakkattu Vasu, Nor Azlin Mohd Nordin, Shazli Ezzat Ghazali

**Affiliations:** a Center for Rehabilitation and Special Needs Studies, Faculty of Health Sciences, Universiti Kebangsaan Malaysia, Kuala Lumpur, Malaysia; b Department of Physiotherapy, Faculty of Medicine and Health Sciences, Universiti Tunku Abdul Rahman, Bandar Sungai Long, Kajang, Selangor, Malaysia.

**Keywords:** anxiety, autogenic relaxation training, physiotherapy

## Abstract

**Introduction:**

The occurrence of post-stroke emotional problems is significant during the early post-stroke stage and affects the recovery of functionality among the survivors. Because stroke survivors require active engagement in rehabilitation to optimize the process of neuroplasticity in the initial stage of stroke, there is a need to integrate an intervention, preferably therapists-mediated during rehabilitation, which reduce emotional problems thus improve motivation level among the survivors. One such technique is autogenic relaxation training (ART). ART has been found to reduce anxiety and depression among patients with several medical conditions. However, its usage in stroke survivors during rehabilitation has been limited to date. Therefore, this study is intended to evaluate the effectiveness of ART in addition to usual physiotherapy in improving emotional state and functional level of stroke survivors during rehabilitation.

**Methods:**

This is an assessor blinded randomized controlled trial comparing 2 intervention approaches namely ART-added physiotherapy (experimental group) and usual physiotherapy (control group). A total of 70 post-stroke patients will be recruited and allocated into either the ART-added physiotherapy or the usual physiotherapy group. The ART-added physiotherapy group will undergo a 20-minute ART session followed by 40 minutes of usual physiotherapy. While the usual physiotherapy group will receive usual physiotherapy alone for 60 minutes. All participants will be treated once a week and are required to carry out a set of home exercises for 2 times per week during the 12-week intervention. Assessment of emotional status and functional independence will be carried out at pre-intervention and week 13 of the intervention with the use of Hospital anxiety and depression scale, Barthel index, and EuroQol-5 dimensions-5 levels. All data will be analyzed using descriptive and inferential statistics.

**Discussion:**

The expected main study outcome is an enhanced evidence-based physiotherapy program that may be used by physiotherapists in the rehabilitation of stroke patients with emotional disturbances.

**Trial registration:**

Australian New Zealand Clinical Trials Registry, ACTRN12619001664134 (last updated on 28/11/2019).

## 1 Introduction

Stroke is one of the disabling conditions among adult and older populations worldwide.^[1]^ Post-stroke rehabilitation, which focus on therapeutic exercises remains the mainstay of treatment aiming to combat post-stroke functional dysfunctions and improve quality of life among stroke survivors.^[2]^ The adherence to the exercises is the key factor of the success in rehabilitation; unfortunately, 65% of stroke survivors fail to adhere to their prescribed exercises due to various reasons including psycho-social problems.^[3–5]^

It is documented that approximately 30% to 45% of stroke survivors have some forms of psycho-social problem such as depression, anxiety, mania, apathy, and fatigue.^[6–8]^ The symptoms may range from relatively minor anxiety to a severe depression. The persistence of these negative consequences often limits the survivors to understand their limitations and post-stroke goals, adopt new skills and adhere to rehabilitation interventions.^[9]^ Post-stroke emotional issues may also influence the working memory performance,^[10]^ mood, and energy levels,^[11]^ and results in negative personality changes such as poor acceptance of disability, poor concentration, being irritable, and unrealistic.^[12,13]^ Eventually, these would affect the post-stroke recovery and quality of life of the survivors.^[14]^

In the past, rehabilitation exclusively focused on motor or movement rehabilitation since it is the most significant problem following a stroke. However, as the mental and psychosocial disability are increasingly recognized as a serious health concern influencing the functionality of the stroke survivors particularly when they return to the community from acute care settings, rehabilitation professionals should now consider these aspects in planning post-stroke activities. Physiotherapists, being the key rehabilitation professional should broaden their roles in post-stroke rehabilitation including recognizing symptoms of mental health and implement suitable strategies to improve emotional state and ensure optimum level of adherence to exercise program.^[3]^

The application of psychological interventions in physiotherapy practice is still at a primitive stage to date.^[2,15]^ Existing physiotherapy practice in stroke rehabilitation shows that emotional factors are not well addressed even though they have been found to negatively impact on daily functioning, quality of life, and functional outcome.^[16,17]^

Relaxation exercises have an important role in reducing the negative effects of the emotional problems arising in many neurology-related conditions such as stroke, cerebral palsy, Parkinson disease, and spinal cord injury.^[18–20]^ There are many available relaxation techniques such as autogenic relaxation training (ART), relaxation–biofeedback, progressive relaxation exercise (PRE), and relaxation—hypnosis. ART is the most commonly used relaxation technique which is found to produce positive effects on various conditions such as anxiety, depression, hypertension, and pain.^[21,22]^ ART is a simple, self- administered exercises with low-cost of implementation which can easily be administered by physiotherapists. To date, very few studies are available on the effects of ART in stroke population.^[23,24]^ The ART when added into a usual physiotherapy could possibly reduce anxiety and provide a positive outlook which may consequently facilitate greater functional independence. The aim of the study is to investigate the effectiveness of the ART when combined with usual physiotherapy in comparison to usual physiotherapy alone. Usual physiotherapy which normally consists of functional training, balance, and gait training is the commonly used therapy for the stroke population.

## 2 Methods

### 2.1 Study design and setting

**Figure 1:**
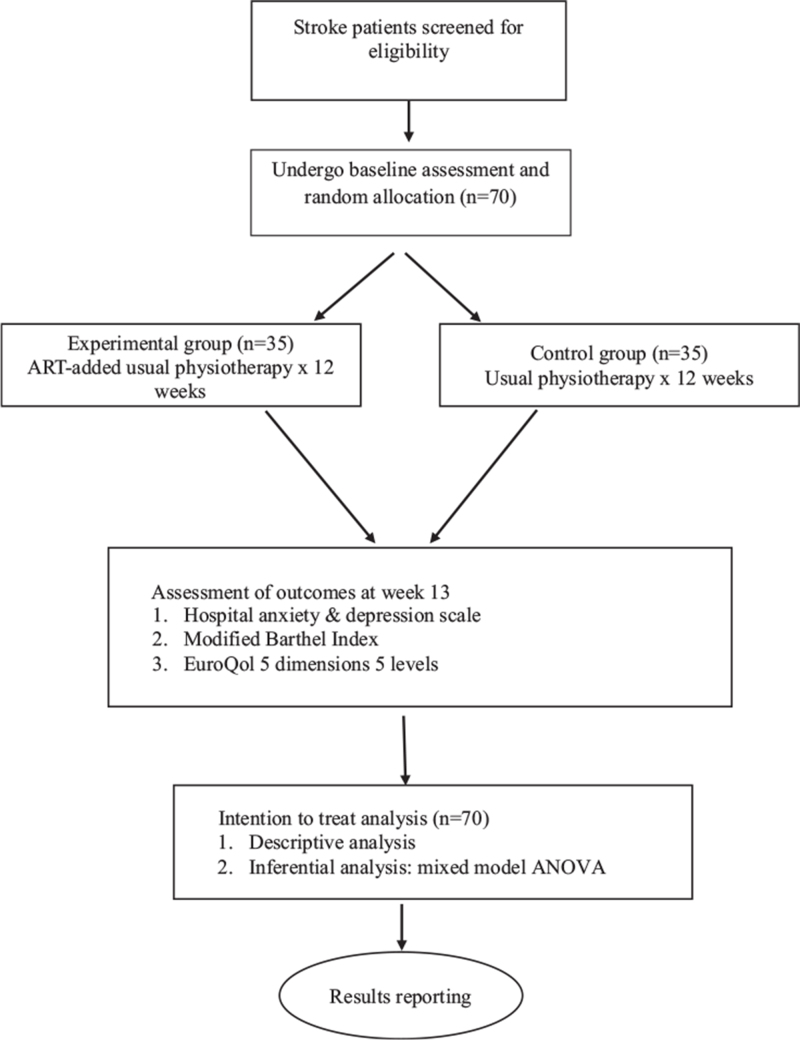
Flow of the study.

A single blind randomized controlled trial will be used as the study design to identify the effectiveness of the ART in addition to usual physiotherapy on post-stroke individuals and comparing this with usual physiotherapy alone. This study will be conducted at Universiti Kebangsaan Malaysia Medical Center, a teaching hospital in the Klang Valley, Malaysia. A one group experimental pilot study will be conducted to identify the feasibility of the ART-added physiotherapy protocol before the main study commences. The flow of the study is shown in Fig. 1.

### 2.2 Study participants

The study participants will be selected with the assistance from physiotherapists in Universiti Kebangsaan Malaysia Medical Centre (UKMMC) using a simple random sampling method from stroke patients’ registry which is maintained by the rehabilitation unit of the hospital.

The inclusion criteria are as follows:

- First-time stroke, diagnosed clinically by a medical physician.
- Sub-acute phase post-stroke (2 weeks–3 months), medically stable condition.
- Age 40 to 75 years.
- Montreal cognitive assessment score ≥26.

The exclusion criteria are:

- Having receptive aphasia.
- History of any psychosomatic disorders.
- Score ≥4 on a modified Rankin scale (wheelchair or bed bound).

### 2.3 Participants allocation

Participants allocation will be conducted by an independent researcher using a stratified randomization method. Sequentially numbered, opaque sealed envelopes will be used to assign the participants into either an experimental group (ART-added physiotherapy) or a control group (usual physiotherapy alone—UP). Stratification variables are age, consisting of adult (40–59 years) and older age (50–75 years), and disability level, namely 1 to 2 (no significant disability or slight disability) and 3 (moderate disability). Therefore, both the ART-added physiotherapy and UP trial arm will consist of subjects with the 2 age and disability levels.

### 2.4 Interventions

The experimental group will receive ART session for 20 minutes, followed by usual physiotherapy for 40 minutes. The ART will be conducted in a quiet room. Participants will be positioned in supine lying or sitting throughout the session and should empty his or her bladder before the session. During the ART session, the participants are asked to loosen any tight clothing, remove watch, spectacles, and stockings. The participants are allowed to close their eyes but not fall asleep while listening to the ART instructions.

Generally, there are 6 standard exercise tasks in ART:

- Tranquility exercise: should create a state of silence and concentration. Typical imagination: “I am calm. Nothing can disturb me.”
- Heaviness exercise: should create a sense of heaviness in the limbs. Typical imagination: “My arms and legs are heavy.”
- Warmth exercise: sense of heat in the limbs. Typical imagination: “My arms and legs are warm.”
- Breathing exercise: concentrated breathing. Typical imagination: “My breath is calm and regular.”
- Heart exercise: Should cause further sedation. Typical imagination: “My heart beats calmly and regularly.”
- Solar plexus exercise: concentration on the celiac plexus should deepen the relaxation. Typical imagination: “The center is warm.”

After the ART session, the participants will be asked to remain on the training mat for 5 minutes before starting usual physiotherapy interventions. Usual physiotherapy in this study consists of general functional exercises, balance, upper limb, and gait training.

The control group will undergo usual physiotherapy alone for 60 minutes. The content of usual physiotherapy is similar to that given to the ART-added physiotherapy group. Blood pressure of each participant will be recorded before and after the intervention session. The participants are instructed to report any unusual feelings that they may experience such as giddiness, nausea, or shortness of breath to the physiotherapist during the intervention sessions.

Both groups undergo the given intervention for 3 times per week (1 supervised and 2 non-supervised) for 12 weeks, for a total duration of 1 hour per session. Monitoring of the participants will be done weekly during the supervised session to ensure adherence to the non-supervised in-home sessions and to assess the occurrence of any adverse effect.

### 2.5 Outcomes

Feasibility of the intervention will be assessed with regards to the participants’ acceptance and adherence, the occurrence of adverse effect and feedback from the participants.

Outcomes of the main study will be measured as follows:

- The anxiety level will be measured using Hospital anxiety and depression scale (HADS), a self-reported questionnaire and most reliable instrument to identify the presence of depression and anxiety in outpatient department.^[25]^ The scoring can be classified as normal (0–7), borderline (8–10), and caseness (≥11).^[26]^ The recommended cut off score for Hospital anxiety and depression scale is ≥8. The tool has established validity and reliability, with a reliability score of 0.88 for anxiety subscale and 0.79 for depression subscale.
- Modified Barthel index (MBI) will be used to measure functional independence of the participants. MBI is a tool to measure the degree of assistance needed by a person, with using 10 specific activities of daily living. Each component is scored by numerical scale and the total score ranges from 0 to 20. The classification is based on physical disability; no physical disability (20), mild impairments (15–19), moderately disabled (10–14), severe disability (5–9), and very severely disabled (0–4). MBI demonstrates high test-retest reliability and inter-rater reliability.^[27,28]^
- EuroQol 5-Dimension 5 Levels (EQ5D5L), a standardized instrument for measuring generic health status will be used to measure quality of life of the participants. The descriptive system comprises 5 dimensions of life namely mobility, self-care, usual activities, pain/discomfort, and anxiety/depression. Each dimension has 5 levels of response: no problems, slight problems, moderate problems, severe problems, and extreme problems. The respondent is asked to indicate his/her health state by ticking (or placing a cross) in the box against the most appropriate statement in each of the 5 dimensions. This response results in a 1-diginumber expressing the level selected for that dimension. The digits for 5 dimensions can be combined in a 5-digit number describing the respondent's health state, termed health utility index. All dimensions of the EQ5D5L are reliable in test–retest with intraclass correlations ranging from 0.67 to 0.81.^[29]^

### 2.6 Assessment of outcomes

Participants will be assessed at baseline and at week 13 of interventions by a therapist who is blinded to the group allocation and trained to conduct the standardized tests. The recorded baseline assessment data will not be accessible to the blinded assessor at post-trial assessment in view to avoid assessment bias.

### 2.7 Sample size

The one group experimental pilot study involves 20 participants which is considered adequate to assess the feasibility of a pilot intervention.^[30]^ For the main study, 70 eligible stroke participants will be recruited. The sample size of 70 was estimated using GPower software and based on mixed-model analysis of variance (ANOVA) test with effect size of 0.34 (Manzoni 2008),^[31]^ significance level *P* < .05 and study power 80%.

### 2.8 Data analysis

All data will be entered into IBM Statistical Packages for Social Sciences (SPSS) version 25.0. Intention-to-treat (ITT) method will be used; all the participants recruited at baseline will be included in the outcome analysis. Using ITT, missing data will be replaced with last observation carried forward. Socio-demography and health profile of the participants will be analyzed descriptively and reported as frequencies (percentages) and mean (standard deviation) or median (inter-quartile range). The effects of the interventions will be analyzed using mixed model ANOVA and reported as main time, group, and time–group interaction effects. The level of significance will be set as *P* < .05 for all results.

### 2.9 Ethics and dissemination

The study received ethical approval (NN-2018–164) from the Ethics committee of the UKMMC, that is responsible to monitor the study progress. All participants will provide an informed consent prior to enrollment in the study. Participants may withdraw anytime during the study without providing an explanation. Participants personal data will be kept confidential, and the study findings will only be published in a peer-reviewed journal.

## 3 Discussion

The main aim of the study is to assess the effectiveness of autogenic relaxation training in addition to usual physiotherapy in comparison to usual physiotherapy alone in reducing emotional disturbances and improve functional level of stroke survivors in the community. Ensuring regular participation of stroke survivors in the rehabilitation is a challenging task and poor participation negatively affects post-stroke recovery. As a facilitator of physical activity, the physiotherapists need to encourage their involvement in rehabilitation and adherence towards the prescribed exercise program. Currently, there is no intervention which has been incorporated in the field of stroke physiotherapy, which influences the emotion and motivation level of stroke survivors.

Although emotional issues are significant post-stroke,^[32–34]^ the use of ART in stroke population has not been well explored. A study conducted on patients with post-stroke anxiety showed significant changes in the HADS after the administration of ART.^[23]^ However, the authors pointed out that the study has many limitations such as small sample size and lack of blinding. Another study conducted to assess self-reported tension in post-stroke patients using tension rating circles also observed potential changes after ART.^[24]^ There were several practical difficulties which emerged during their study especially in ART session management, staffing resources, and awareness of attendance criteria. Nonetheless, the participants reported greater self-motivation after the ART session.

The 2 studies^[23,24]^ considered mainly the effectiveness of ART on post-stroke anxiety. Changes in motor performance as a result of psychological improvement among the stroke survivors following ART were not studied. This proposed study will analyze the effect of physiotherapist-mediated ART on anxiety and functional independence. The results of the study will provide the information on the functionality and quality of life among those who has anxiety and how this change following ART and physiotherapy. The study will also assess the feasibility of setting up a relaxation group protocol to handle post-stroke anxiety in an out-patient rehabilitation setting. This will help to fill the gap in the field of ART usage on mental state and motor performance among stroke population.

We anticipate that the stroke survivors who receive ART in addition to usual physiotherapy would report reduction in their anxiety level, and improvement in functional ability and quality of life. We also predict that the combined intervention would be feasible and may be introduced as a strategy to enhance post-stroke rehabilitation which is currently under-optimized.^[35,36]^

## Acknowledgment

The authors thank the Research and Ethics Committee of Universiti Kebangsaan Malaysia (UKMMC) for the study approval.

## Author contributions

**Conceptualization:** Nor Azlin Mohd Nordin, Deepak Thazhakkattu Vasu, Shazli Ezzat Ghazali.

**Funding acquisition:** Nor Azlin Mohd Nordin.

**Methodology:** Nor Azlin Mohd Nordin, Deepak Thazhakkattu Vasu.

**Supervision:** Nor Azlin Mohd Nordin, Shazli Ezzat Ghazali.

**Writing – original draft:** Deepak Thazhakkattu Vasu.

**Writing – review & editing:** Nor Azlin Mohd Nordin.
